# Ethical aspects of malaria control and research

**DOI:** 10.1186/s12936-015-1042-3

**Published:** 2015-12-22

**Authors:** Euzebiusz Jamrozik, Vânia de la Fuente-Núñez, Andreas Reis, Pascal Ringwald, Michael J. Selgelid

**Affiliations:** Centre for Human Bioethics, Monash University, Clayton, VIC Australia; Department of Knowledge, Ethics and Research, World Health Organization, Geneva, Switzerland; Global Malaria Programme, World Health Organization, Geneva, Switzerland

## Abstract

Malaria currently causes more harm to human beings than any other parasitic disease, and disproportionally affects low-income populations. The ethical issues raised by efforts to control or eliminate malaria have received little explicit analysis, in comparison with other major diseases of poverty. While some ethical issues associated with malaria are similar to those that have been the subject of debate in the context of other infectious diseases, malaria also raises distinct ethical issues in virtue of its unique history, epidemiology, and biology. This paper provides preliminary ethical analyses of the especially salient issues of: (i) global health justice, (ii) universal access to malaria control initiatives, (iii) multidrug resistance, including artemisinin-based combination therapy (ACT) resistance, (iv) mandatory screening, (v) mass drug administration, (vi) benefits and risks of primaquine, and (vii) malaria in the context of blood donation and transfusion. Several ethical issues are also raised by past, present and future malaria research initiatives, in particular: (i) controlled infection studies, (ii) human landing catches, (iii) transmission-blocking vaccines, and (iv) genetically-modified mosquitoes. This article maps the terrain of these major ethical issues surrounding malaria control and elimination. Its objective is to motivate further research and discussion of ethical issues associated with malaria—and to assist health workers, researchers, and policy makers in pursuit of ethically sound malaria control practice and policy.

## Background

Malaria morbidity and mortality are largely preventable via existing interventions (e.g., anti-malarial drugs, insecticide, nets, and other vector control measures). There has been a significant increase in financial aid directed at malaria control in the last decade, resulting in a halving of global malaria burden since 2004 [1, 2]. However, of the approximately US $5.1 billion required for malaria control initiatives in 2013, only $2.8 (i.e., 55 %) was available from existing funding mechanisms (from individual countries and international sources combined) [3], meaning that many people do not even benefit from the simple protection of inexpensive and cost-effective bed nets [1]. Furthermore, access to such interventions is often inversely proportional to wealth and proximity to major urban centres, and can be especially poor for mobile populations at high risk of malaria [4, 5].

Programmes combatting other similarly important infectious diseases, such as HIV and tuberculosis, raise many ethical issues that have been identified and addressed to some extent [6–8], yet malaria control, treatment and research have received comparatively little explicit ethical analysis and debate. This article summarizes the limited existing literature on ethical issues associated with malaria and highlights areas in need of further work. Though some scientists or health professionals might have a narrow conception of ethics as a process of research review, ethics more generally is broadly concerned with questions about value, what actions ought to be taken, and what policy should be. Such questions partly turn on the aim to achieve good outcomes—such as maximal disease burden reduction–but they also concern issues of justice and the moral obligations of medical professionals and patients. With this in mind, this review maps the terrain of such issues in the context of malaria control, offers some preliminary analyses, and draws attention to matters requiring further reflection and/or research.

### Epidemiology and justice

The prevalence of malaria is inversely proportional to GDP per capita. Sachs and Malaney aptly note that “where malaria prospers most, human societies have prospered least” [9]. Vulnerability to malaria, like many other infectious diseases, is largely a product of social determinants of health—such as poverty, malnutrition, and insufficient access to healthcare. Like HIV and other diseases of poverty, malaria is not only an effect of poverty but also further impoverishes families and communities [9, 10].

International efforts towards malaria control thus represent a major opportunity to remedy some of the past and present injustices manifested by global disparities in wealth and freedom from preventable disease [11, 12]. *Inter alia*, justice requires both improvement of access to existing interventions and increased research and development of new tools for malaria control, both of which require an influx of funding from wealthy nations and donor organisations. Malaria burden is a major example of global inequities, which are receiving heightened attention at the international level as reflected by the Sustainable Millennium Development Goals.

### Climate change

Anthropogenic climate change affects human health in many ways, and has the potential to alter malaria disease patterns by changing the habitats of *Anopheles* mosquitoes, and by displacing human populations [13–15]. Areas potentially at increased risk of malaria include high altitude (>1,600 m) areas, especially in East Africa [16] and potentially temperate zones in central Asia, Europe and North America [17]. While future predictions are necessarily uncertain, the multiple mechanisms by which climate influences malaria warrant further research. An increase in malaria burden due to climate change is also a matter of justice, since those who would suffer most are (usually/often) those who (1) are already among the worst-off groups of (global) society and (2) themselves contribute least to climate change. The nexus of climate and disease provides additional reasons to minimize climate change (to prevent disease spread), and conversely to intervene on malaria transmission in the present, since this will help mitigate any future relative increase in malaria risk due to climate change. Similar reasoning applies to the wide range of other diseases (including dengue) which may be influenced by the same mechanisms.

### Access to malaria control and the rise of resistant malaria

A core ethical issue for malaria policy is the urgent need for universal access to control interventions [3, 5]. With regards to prevention, the use of long-lasting insecticide-treated bed nets is a cost-effective way to reduce the disease burden of malaria in communities in endemic areas, although effectiveness varies by region and widespread coverage is needed for greatest benefit [18]. Since nets protect not only those who sleep under them but also the wider community by reducing transmission, a community-level benefit is placed at risk when a significant proportion of families cannot afford even subsidized nets due to severe poverty. Everyone nearby benefits when all have access to nets, thus there is a moral imperative that bed nets (like many vaccines) are universally available and, where necessary, free of charge [19, 20].

The cost of anti-malarial medications poses similar challenges, and lack of access to adequate health services commonly leads to treatment failure and death. Counterfeit drugs, for example, may be more widely available in areas with limited healthcare infrastructure [21]. In addition to having direct adverse effects on patients, counterfeit drugs contribute to the emergence of drug resistance [22].

Overall the lack of access to prevention and treatment for the most impoverished or isolated will only increase the injustices associated with malaria. Universal coverage that is free or genuinely affordable will be the only way to sustainably control or eradicate the disease. It will also have secondary benefits such as decreasing pressure on hospitals by reducing the total incidence of severe malaria, and by preventing the emergence and spread of resistance.

### Anti-malarial resistance

Anti-malarial resistance, especially to artemisinin-based combination therapy (ACT), is a major threat to malaria control efforts. Resistance generally results from inappropriate, incomplete or inadequate courses of treatment. This most commonly occurs in the context of poverty, counterfeit medications, and weak healthcare infrastructure [1, 23]. The major early ‘hotspots’ for ACT resistance were clustered around national border zones in the Greater Mekong sub-region of South East Asia. The presence of impoverished, mobile and politically marginalized populations, including large numbers of refugees, are likely contributors to this phenomenon [24, 25]. As with drug resistance in other diseases, political and socioeconomic factors are clear drivers of anti-malarial resistance, which is thus an issue of international justice. In addition, wealthy countries should be concerned about anti-malarial resistance for self-interested reasons, as (i) resistant strains of malaria may spread beyond the borders of poor countries and (ii) the economic costs of control efforts will rise with increasing resistance.

The current ethical priorities to address drug resistance are therefore: universal availability of (i) diagnostic support (including resistance testing), (ii) targeted, locally effective treatments, before multi-resistant strains emerge, and (iii) increased research and development of new drugs (to combat multi-resistant strains). Universal access to these resources would significantly address current injustices, and help to prevent major future harms and increased costs due to resistance.

### Individual and public health interests

Several malaria control strategies have specific ethical implications. The following sections address some examples that require the balancing of individual liberties against public health goals.

### Mandatory screening

Mandatory screening has been implemented in many countries targeting or maintaining malaria elimination. This involves the compulsory testing of asymptomatic travellers or immigrants from malaria endemic zones [26, 27]. There is a strong ethical rationale for mandatory screening for malaria in such circumstances. First, the individual being screened stands to benefit from treatment if infected. Second, significant public health risks may be averted by preventing new outbreaks [28]. Importantly, the minimal limitations on individual liberty imposed by mandatory screening should be weighed against the significant individual and community benefits.

Resistant malaria creates a further justification for mandatory screening e.g., to prevent the spread of ACT-resistance from South East Asia to India and Africa [29]: a recent example is the screening of Cambodian peacekeeping troops in 2014 before deployment to Mali. In all cases, a clear ethical framework should be developed to inform screening programmes, and the testing used should be chosen with care. The number of irregular migrants and refugees from endemic countries can increase the practical and ethical complexity of such policies [30]; implementation should aim at benefits for both migrants and local communities, without creating undue restrictions, discrimination, or stigma for those from high prevalence areas [31].

### Mass drug administration

At present, the circumstances in which mass drug administration (MDA) would provide a cost-effective long-term benefit have yet to be clearly defined, but it could play an important role in eradication efforts [32]. Ethically, it is important to consider the fair distribution of benefits and burdens, noting that infected individuals will receive the greatest immediate benefits while all (including those not infected) may share the risks and burdens of treatment, including increased resistance where MDA does not achieve elimination. Individual and community benefit would be highest in seasonal prophylaxis, although there are potential issues with delaying natural immunity, and thus a rebound in incidence and severity. Conversely, elimination efforts may be most effectively targeted at populations and times with relatively low levels of parasitaemia, where the balance of risks and benefits requires careful consideration, noting the benefits of elimination to both current and future generations.

In all cases, communities should be closely involved in programme design. If mass screening is used prior to MDA, the test should be chosen based on programme goals and community acceptability where feasible, some form of informed consent at the community and/or individual level should be coupled with well-designed education and social awareness campaigns to ensure population-wide effectiveness [33].

### Primaquine

The aminoquinolones (primarily primaquine) present a unique ethical case in malaria control. The use of such drugs is one of the few ethical issues specific to malaria that has received explicit ethical analysis [34]. When used in low-doses as a gametocidal agent for *Plasmodium falciparum*, primaquine is safe and largely effective [35], yet higher doses used in anti-relapse therapy for *Plasmodium vivax* carry higher risks. While treatment-related deaths are rare, the risks of haemolysis conferred by different genotypes and phenotypes in different populations have not been well defined, and no point-of-care test can determine individual risk. This poses practical as well as ethical challenges for clinicians and policy makers, especially where primaquine is considered in *P. vivax* elimination MDA including aparasitaemic individuals, for whom the ratio of risks to benefits would be highest [34].

Alternative agents are needed for those who cannot take primaquine, including pregnant women [34]. In the absence of alternatives, appropriate treatment with primaquine must continue, especially in regions with a high burden of *P. vivax.* Meanwhile, the moral priorities to adequately inform patients and reduce overall harms where possible should guide further research aimed at improving treatment programmes. Thus, ultimate goals of research in this area should be development of (i) accurate point-of-care testing for risk of haemolysis (G6PD deficiency) and (ii) less toxic alternatives to primaquine. Current efforts should meanwhile include (i) epidemiological mapping of G6PD deficiency traits in regions aiming at elimination, (ii) further elucidation of phenotypic risk in different genotypes of G6PD deficiency, and (iii) primaquine dose-finding studies [34, 36–38].

### Blood donation and transfusion

Malaria was one of the first recorded transfusion-transmitted infections [39]. Although the burden and risk of transfusion-transmitted malaria is low in comparison to infections such as HIV and viral hepatitis [40], the issue of transfusion-transmitted malaria has been largely overlooked. This has resulted in inconsistent practices and policies regarding donor screening, disclosure of disease status, and provision of treatment or preventative measures to donors and recipients [41]. For instance, in some areas all blood donors are screened for malaria whereas, in others, treating all blood donors with anti-malarials is common practice [39]. Ethical analysis of benefits and risks should underpin the development of relevant guidelines. For example, robust disclosure and treatment mechanisms should be put in place to benefit asymptomatic donors who are diagnosed with malaria via blood screening [42].

### Ethical issues in malaria research

#### Controlled infection studies

Controlled infection studies involve intentionally exposing human beings to pathogens in order to observe the natural history of disease, develop research models of infection, or test the efficacy of drugs and vaccines [43]. Modern studies in controlled malaria infection date to 1900 [44] and continued during World War II [45, 46]. Current studies are performed safely, with no deaths or *lasting* symptoms among participants [47]. However, participants often experience significant *acute* symptoms—including fever, headache, joint and muscle pains, and even cardiac events—which go beyond many definitions of ‘minimal harm’ that are commonly employed in the ethical assessment of non-therapeutic research [43]. Further, malaria infection studies often involve exposure to blood products via mosquitoes or injection [48], which poses the risk of transmitting other infections and prion diseases. Finally, if participants are able to leave research centres while still infected, this can pose risks to the wider community. In all cases, participants must be carefully selected and informed.

Yet such studies remain important in testing new vaccines or treatments, and it may be more useful to perform them in malaria endemic populations. Risks of controlled infection studies may be somewhat less for participants with substantial immunity from prior infection, and the benefits of participation may also be greater (e.g., in the case of studies testing new vaccines). On the other hand, such studies should only occur in contexts with access to affordable quality healthcare, high standards of research conduct, and robust ethical oversight so that the safety of participants is ensured [49].

#### Human landing catches

Malaria research to measure mosquito or malaria density has often involved the use of human landing catches, where human participants act as mosquito ‘traps’. In some cases, people working as human landing catches may receive 50–100 mosquito bites in one nightshift [50]. The use of human landing catches poses similar ethical issues to those raised by controlled infection studies, where potential harms to a few individuals are (voluntarily consented to and) balanced against benefits to the wider community. In practice, the absolute harm appears to be low, since people undertaking this kind of work are provided with a high standard of diagnosis and treatment [43, 47]. These studies have provided essential insights into the biology and transmission of malaria that may not have been feasibly obtained by other means. It would nonetheless be ethically preferable to develop alternative practices that offer no risk to human beings. In this context, trials of novel mosquito traps [51] are a welcome development, though human landing catches remain a ‘gold standard’ against which these traps are tested [52].

#### Transmission-blocking vaccines

Ethical issues related to vaccination have been discussed for other diseases [53, 54], and should be considered in both the investigation and eventual implementation of malaria vaccines. For example, the success of the RTS,S vaccine (45 % vaccine efficacy in children aged 5–17 months and 34 % in infants, with some protection against severe malaria in children but not infants [55]) raises the question of what trials of other vaccines should use as an ethically acceptable control arm (placebo or RTS,S). Requirements such as those of the Declaration of Helsinki that all new agents be tested against the standard of care are controversial in this context and, with careful consideration, an argument could be made to support using placebo despite the existence of a partially effective vaccine [56].

The avenue of transmission-blocking vaccines (TBVs) is a novel case study in vaccine ethics, especially since they aim only at preventing transmission of malaria from the vaccinated person to healthy individuals [49]. Though there are indirect self-interested reasons to be vaccinated (protecting one’s family and reducing intra-household transmission), individuals would primarily be administered a TBV in order to create effective herd protection. Importantly, herd protection also extends to those who do not respond to, or cannot safely receive, vaccines, and is the main mechanism by which such individuals can benefit from vaccination [53]. In such cases a substantial majority of the population needs to be vaccinated, and ethical approaches to collective vaccination have been published elsewhere [54]. Where the vaccine is proven to be safe and effective and the public health goal is sufficiently important (e.g., the eradication of malaria), there are likely to be strong ethical grounds for universal vaccination. Thus, effective vaccination infrastructure covering the entire target population as well as education and social campaigns to ensure high levels of uptake would be of paramount importance in any eventual implementation of TBVs. Meanwhile, TBV researchers should consider whether to co-administer other interventions (perhaps even other vaccines) with TBVs in trial settings in order to optimize individual benefit.

#### Genetically-modified (GM) mosquitoes

Multiple strategies for genetic modification of mosquitoes are under study, aiming to contribute to transmission interruption for malaria (and other mosquito-borne diseases) [57–59]. Implementation of such strategies depends both on adequate scientific development and acceptance of local communities. Currently, the ecological consequences of GM mosquitoes are poorly understood [60]. Although risks will vary with different techniques, they would often be difficult to control once such insects were released. This raises ethical questions about how to balance potential significant improvements in population health against possible environmental harms, and whether other interventions with fewer risks would be preferable. Where multiple GM techniques are available, the relative risks of each should be considered. Policies regarding the use of GM mosquitoes should be informed by ethical analysis and prospective consultation at the level of research groups, local communities, national governments and international organisations. Groups planning to release GM mosquitoes have the strongest obligations towards communities who will be most affected, and strategies to ensure responsible practice have been published by WHO [61].

## Conclusions

Ethical issues in malaria control and research have hitherto received little explicit analysis in the published literature. This review provides an initial summary of the main issues at hand, but further work is required in many of the areas discussed above, especially since the field is likely to evolve with future changes in malaria science, epidemiology and policy.

The epidemiology of malaria is a stark reminder of global injustice in relation to disease risk and health outcomes. The recent improvements in funding and control initiatives have in part been driven by an awareness of these injustices: that those least able to access malaria control mechanisms are often those who need them most, and that there are significant research gaps for malaria and other diseases of poverty. Similarly, ethics can help to guide policy and treatment in areas such as the control of anti-malarial resistance, mass screening and treatment programmes, and the use of primaquine. In malaria research, specific ethics guidelines should be developed to ensure valuable research programmes continue without imposing excessive risks on participants. Looking toward future possibilities, prospective consideration of ethical issues may assist in the appropriate and successful implementation of novel techniques such as malaria vaccines or genetically-modified mosquitoes. Recent capacity building efforts for local ethics expertise in endemic regions have been a positive development.

The preliminary analyses offered here are intended to motivate further research on ethical issues associated with malaria, inform ethics review processes related to control interventions, and assist health workers, researchers, and policy-makers in pursuit of ethically sound malaria control efforts. The ultimate beneficiaries of such work will include patients and residents of malaria endemic areas who are in the greatest need of ethically designed malaria programmes now and in the coming decades.
